# Social Withdrawal Behaviour at One Year of Age Is Associated with Delays in Reaching Language Milestones in the EDEN Mother-Child Cohort Study

**DOI:** 10.1371/journal.pone.0158426

**Published:** 2016-07-08

**Authors:** Antoine Guedeney, Anne Forhan, Beatrice Larroque, Maria de Agostini, Jean-Baptiste Pingault, Barbara Heude, EDEN mother-child cohort study group

**Affiliations:** 1 Univ Denis Diderot Paris - Cité & INSERM UMRS 1178, 94807, Villejuif, France; 2 INSERM, UMR1153 Epidemiology and Biostatistics Sorbonne Paris Cité Center (CRESS), Early ORigin of the Child's Health and Development Team (ORCHAD), Paris Descartes University, Paris, F-75014, France; 3 Epidemiology and Clinical Research Unit, Beaujon Hospital, Clichy, 92110, France; 4 Division of Psychology and Language Sciences, University College London, WC1E6BT, London, United Kingdom; 5 King’s College London, MRC Social, Genetic and Developmental Psychiatry Centre, Institute of Psychiatry, WC1E6BT, London, United Kingdom; Hôpital Robert Debré, FRANCE

## Abstract

**Objective:**

The aim of the study was to examine the relationship between social withdrawal behaviour at one year and motor and language milestones.

**Materials and Methods:**

One-year old children from the EDEN French population-based birth cohort study (Study on the pre- and postnatal determinants of the child’s development and prospective health Birth Cohort Study) were included. Social withdrawal at one year was assessed by trained midwives using the Alarm Distress BaBy (ADBB) scale. Midwives concurrently examined infants’ motor and language milestones. Parents reported on child’s psychomotor and language milestones, during the interview with the midwife.

**Results:**

After adjusting for potential confounding factors, social withdrawal behaviour was significantly associated with concurrent delays in motor and language milestones assessed by the midwife or the parents.

**Discussion:**

Higher scores on social withdrawal behaviour as assessed with the ADBB were associated with delays in reaching language milestones, and to a lesser extent with lower motor ability scores. Taking the contribution of social withdrawal behaviour into account may help understand the unfolding of developmental difficulties in children.

## Introduction

A key element in early development is the ability to synchronize communication within the parents-infant triad, particularly during the first 18 months of the infant’s life [1, 2]. Social withdrawal behaviour in infants is characterized by less frequent positive behaviours e.g. eye contact, smiling, cooing or by negative behaviours, as self-stimulation [3]. Importantly, social withdrawal is a normal feature of infant behaviour in parent-infant interactions, providing a way for the infant to regulate the flow of interaction [4, 5]. However, at a clinical level, sustained social withdrawal is recognized as a defence mechanism when the child is faced with adverse situations he or she cannot cope with. Infant withdrawal seems to be a key element of the infant’s response in the face of parent-infant relationships lacking in synchronization, with repeated failure to repair the relationship mismatches [2, 4, 5]. Sustained withdrawal behaviour may stem from relationship disorders, as the infant’s ability to relate to his or her social world develops within close and continuous interactions between parents and infant. Social withdrawal behaviour may also stem from temperamental/genetic susceptibility [6], sensory integration disorders, or from a set of impairments and disabilities, e.g. very low birth weight and prematurity [7]. Regardless of the origin—the child and/or his relational environment—excessive withdrawal from social interactions is a robust marker of infant distress [4, 5].

Because excessive social withdrawal disrupts the ability of infants to adequately interact with caregivers, parents or others, it may in turn undermine the normal course of development [4, 8]. As such, we expect socially withdrawn children to also experience difficulties in psychomotor and language development. The present study aims to examine how social withdrawal behaviour in infants correlates with some delays in psychomotor and language milestones, using the data from the EDEN study, a population-based birth cohort [9].

## Materials and Methods

### Participants

**Fig 1:**
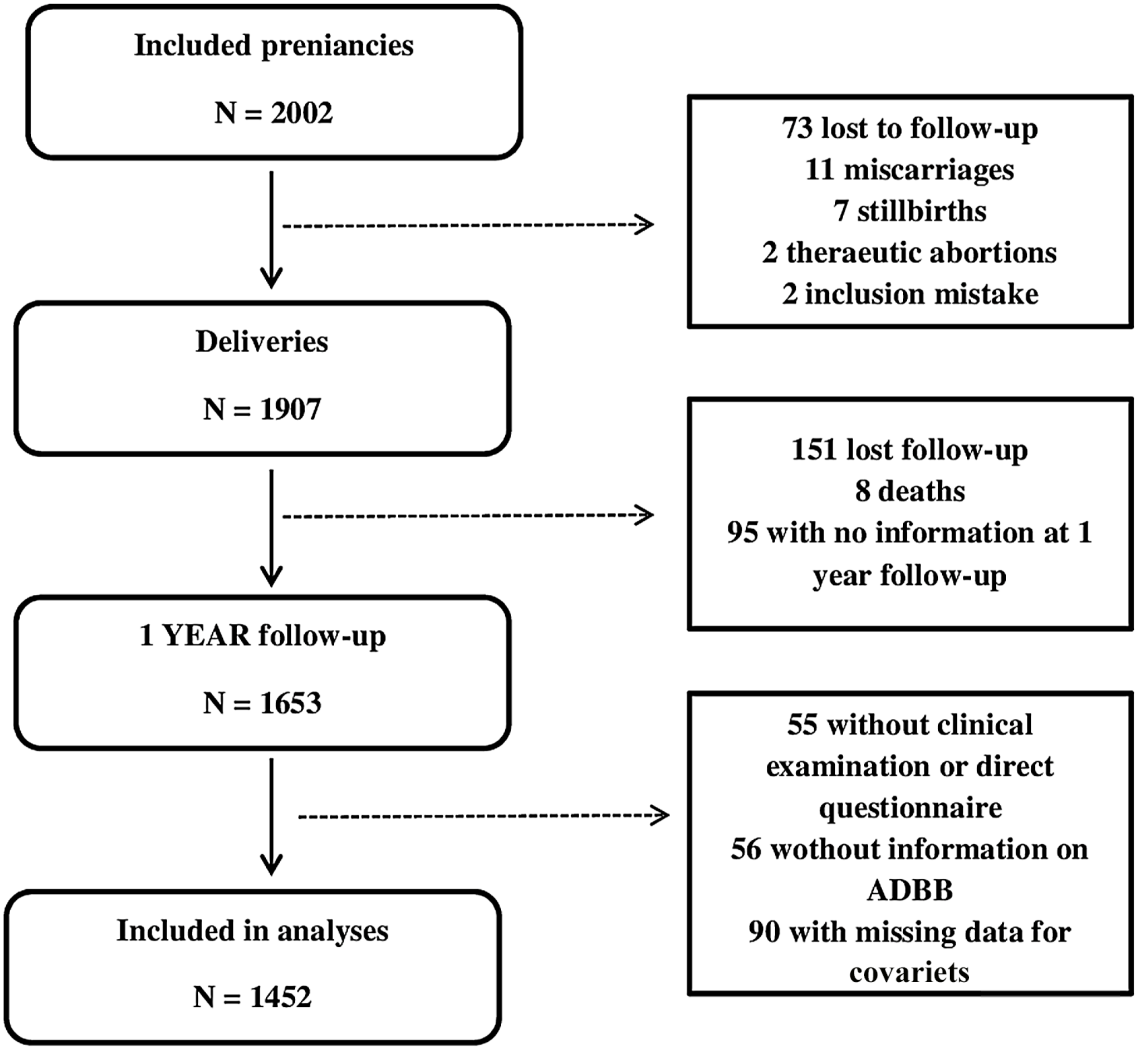
Flow chart for study population. The EDEN Mother-Child Cohort.

Participants of the EDEN mother-child birth cohort study [9] were recruited between 2003–2006 among pregnant women (24 weeks of amenorrhea) followed in two maternity wards in Poitiers and Nancy University hospitals (France). Exclusion criteria were multiple pregnancies (e.g. twins), diabetes diagnosed prior to pregnancy, illiteracy or moving outside the region in the following 3 years. Among women who fulfilled the inclusion criteria, 53% agreed to participate. A total of 2,002 women were included in the study, and 1907 were still followed-up at delivery (Fig 1). At age one year, 1598 infants of the EDEN study were examined, by a trained team of midwifes. Of these, 1452 had a complete ADBB assessment and had no missing data for covariates.

The study was approved by the Ethics Committee of Kremlin-Bicêtre hospital and by the French Data Protection Authority. Written consents were obtained from the mother.

### Measures

When their child was one year old, parents were sent a questionnaire and were invited to an assessment. The direct assessment of the child’s development milestones was conducted by a midwife and consisted of an interview with the mother as well as a direct physical examination of the child.

#### Social withdrawal behaviour at age 1 year

Social withdrawal behaviour was assessed with the Alarm Distress Baby scale [3] designed to assess social withdrawal behaviour in children aged between 2 and 24 months, in the context of routine paediatric examinations or during specific psychological assessments. To facilitate the observation of the child’s behavioural reactions, the observer, here a midwife, engages the child in social interactions through talking, touching and smiling. The scale comprises eight items including: lack of facial expression; lack of eye contact; lack of general level of activity; presence of self-stimulating gestures; lack of vocalizations; lack of rapidity of response to stimulation; lack of relationship with the observer; lack of attractiveness to the observer. Each item is rated from 0 to 4 resulting in 0 as the minimum and 32 as the maximum ADBB total score. The higher the ADBB score, the greater the signs of social withdrawal displayed by the infant. Validation studies of the ADBB in different countries have shown that infants with scores of 5 or above are at risk of adverse outcomes [5]. Reliability of ADBB scores in the EDEN-ADBB study has been reported previously and was achieved by the 2 midwifes through training with the first author through video clips, until a 0.8 Cohen’s kappa inter-rater reliability score was reached). [10].

### Outcomes

#### Psychomotor and language outcomes

Assessment of psychomotor and language was made by a direct examination by a specifically trained midwife (called midwife assessment), and by a questionnaire addressed to the parents during the clinical examination. Items were taken from the second version of the French Developmental Test Brunet-Lezine II by Josse [11] and from the Bayley Scales of Infant Development [12]. Prior to the study, midwives were trained regarding the use of these developmental tests, and milestones of normal language and psychomotor development.

Scores were built by summing up the different binary items within a same domain (motor ability, coordination, language) from the midwife or the parental assessment. When only one or two items of the score were missing for a child, the score was computed using mean imputation. We chose a threshold for each score in order to categorize lower developmental level vs. others. This threshold was based on the 20th percentile of the distribution of each composite score, in order to keep a sufficient number of children in the groups, and preserve statistical power in the analyses. For two scores (motor ability assessed by the midwife, and coordination parental questionnaire) however, the 20th percentile was not specific enough (i.e. including actually more than 50% of children, due to a high number of *ex-aequo*), and the 10th percentile was chosen instead.

#### Confounding variables

During the first visit performed between 24–28 weeks of amenorrhea, mothers declared their date of birth and the highest diploma obtained. Smoking status (no / < 10 cig per day / ≥ 10 cig per day) and alcohol consumption during pregnancy (no / yes) were determined from the questionnaires filled by the mother during pregnancy and at delivery [9]. During mother’s pregnancy, fathers also reported the highest obtained diploma, and then we determined parental education as the average years of education of mother and father. From obstetric and paediatric records, we extracted data on maternal hospitalisation during pregnancy, birth weight, offspring’s gender and gestational age at delivery. In order to include independently birth weight and gestational age at birth in the multivariate models, birth weight was used as a z-score from Gardosi’s & al equation [13] taking into account gestational age and gender. At the first year visit, mothers reported child’s day care arrangements: mother, family (father, grandparents), nursery or other (child-minder, neighbour). The duration of exclusive breastfeeding was calculated using child’s food records assessed at 4 and 8 month after birth [14]. Maternal depressive symptoms were evaluated through self-administered questionnaire one year after birth using the Edinburgh Postnatal Depression Scale (EPDS) [15, 16, 17].

#### Statistical analysis

Univariate tests were first used to investigate the relation between:

1. The ADBB total score and the developmental outcomes (language and motor assessments)
2. The ADBB total score and the adjustment factors (i.e. child and maternal characteristics).
3. Adjustment factors and the developmental outcomes.

Populations included or not in the analysis were also compared. Chi-square and Student test were used for categorical and continuous variables respectively. Non-parametric exact Fisher tests were used to compare percentages when considering isolated items.

A multivariate logistic regression was then used to predict each motor and language outcome (scores over the selected threshold). The ADBB total score and the potential confounding variables were entered in the regression as independent variables. Potential adjustment factors were selected as previously shown to be associated with the ADBB score in the EDEN population (unadjusted p-value <0.20) [18, 19] or showing an association with one of the studied developmental outcome (unadjusted p-value <0.20). In order to have the same set of adjustment factors in the multivariable models of all outcomes, factors were maintained in all the models when associated with the outcome in at least one of them (p<0.20).

Several sensitivity analyses were conducted in order to check the robustness of the results. First, the midwives could report whether they had some difficulties during the clinical examination because of how the baby behaved. Some difficulties, such as cries, sleepiness, illness, fever, tiredness, etc. were indeed reported for 51 infants. We performed the analyses excluding these children. We also performed the analyses excluding children born prematurely (<37 weeks of gestation).

All statistical analyses were performed using SAS 9.3 software (SAS Institute Inc., Cary, North Carolina).

## Results

Comparisons of maternal and children characteristic between children included or not in the analysis are displayed in S1–S6 Tables. About 20% of the children were considered as socially withdrawn, with total ADBB scores at 5 and over. As displayed on Table 1, centre, gestational age, birth weight, hospitalisation during pregnancy, child care, maternal depression at 1 year and parental education differed significantly according to social withdrawal status.

**Table 1:** Maternal and infant characteristics according to social withdrawal at 12 months. The EDEN Mother Child Cohort.

	All N = 1452	ADBB < 5 N = 1163	ADBB ≥ 5 N = 289	p
Centre (Nancy)	740 (51)	614 (52.8)	126 (43.6)	0.005
Sex: Male	767 (52.8)	616 (53)	151 (52.2)	0.83
Exact age of the child at examination (days)	370.1 ± 0.3	370.4 ± 0.3	369.3 ± 0.7	0.16
Length of gestation (weeks)	39.3 ± 0	39.3 ± 0	38.9 ± 0.1	0.0002
Birth weight (g)	3290 ± 13	3306 ± 15	3224 ± 30	0.014
Maternal age at delivery (years)	29.8 ± 0.1	29.8 ± 0.1	29.7 ± 0.3	0.90
Hospitalisation during pregnancy (days)	1.3 ± 0.1	1.2 ± 0.1	2 ± 0.3	0.006
Breastfeeding duration (months)	3.4 ± 0.1	3.4 ± 0.1	3.3 ± 0.2	0.68
Main day time caregiver:				
Nursery	169 (11.6)	144 (12.4)	25 (8.7)	0.026
Other	606 (41.7)	486 (41.8)	120 (41.5)	
Family	136 (9.4)	117 (10.1)	19 (6.6)	
Mother	541 (37.3)	416 (35.8)	125 (43.3)	
Maternal depression scale score (EPDS) at 1 year:				
Unknown	104 (7.2)	78 (6.7)	26 (9)	0.041
< 10	1144 (78.8)	932 (80.1)	212 (73.4)	
≥ 10	204 (14)	153 (13.2)	51 (17.6)	
Maternal alcohol intake during pregnancy (Yes)	642 (44.2)	508 (43.7)	134 (46.4)	0.411
Maternal smoking during pregnancy (cigarettes/day):				
0	1108 (77.7)	891 (77.9)	217 (77)	0.096
1–9	267 (18.7)	218 (19.1)	49 (17.4)	
≥ 10	51 (3.6)	35 (3.1)	16 (5.7)	
Parental education* (years):				
> 12	894 (61.6)	732 (62.9)	162 (56.1)	0.031

Numbers are N (%) or m ± SD

* Calculated as the average of father’s and mother’s years of education

Concerning child development at one year, all motor or language competencies assessed by the midwife were less frequently acquired in children with an ADBB score of 5 or more (Table 2).

**Table 2:** Psychomotor and language outcomes as observed by midwives at age 1 year, according to social withdrawal. The EDEN Mother-Child Cohort.

	N	All N = 1152	ADBB < 5 N = 1163	ADBB ≥ 5 N = 289	p^a^
***Motor ability***					
Walks: *no*	1429	1149 (80.4)	897 (78.4)	252 (88.4)	< .0001
Child can hold sitting: *no*	1447	4 (0.3)	0 (0)	4 (1.4)	0.0015
Child can hold standing: *abnormal*	1427	96 (6.7)	58 (5.1)	38 (13.4)	< .0001
Holds a cube: *no*	1440	16 (1.1)	8 (0.7)	8 (2.8)	0.007
Holds a piece of cereal: *no*	1421	16 (1.1)	6 (0.5)	10 (3.5)	0.0002
***Language***					
Child says different syllables: *not observed*	1452	464 (32)	290 (24.9)	174 (60.2)	< .0001
Child says ‘Mom’ or ‘Dad’: *not observed*	1445	1095 (75.8)	833 (72.1)	262 (90.7)	< .0001
Child says 2 different words with same syllables; other than ‘Mom’ or ‘Dad’: *not observed*	1449	908 (62.7)	664 (57.2)	244 (84.4)	< .0001
Child says a word with 2 different syllables: *not observed*	1449	1267 (87.4)	991 (85.4)	276 (95.8)	< .0001
Child says several different words: *not observed*	1441	1366 (94.8)	1083 (93.8)	283 (98.6)	0.0005

^*a*^ *Exact Fisher’test*

As shown in Table 3, mothers answered most frequently “not yet” when their child presented social withdrawal, to midwives’ questions on what their child was able to do, with few exceptions (whether the child could stand up without help, whether he pronounced different syllables, and for questions on motor coordination).

**Table 3:** Psychomotor and language milestones at age 1, as answered by parents to midwives questions, according to social withdrawal. The EDEN Mother-Child Cohort.

	N^a^	All N (%)	ADBB < 5 N = 1163	ADBB ≥ 5 N = 289	p^b^
***Global motor ability***					
Child walks alone: *not yet*	1451	1102 (75.9)	866 (74.5)	236 (81.7)	0.011
Child alone can stand a few seconds with aid: *not yet*	1117	89 (8)	60 (6.8)	29 (12.1)	0.010
Child alone can sit up from a lying position: *not yet*	1117	170 (15.2)	119 (13.6)	51 (21.3)	0.004
Child alone can stand up with aid: *not yet*	1117	244 (21.8)	166 (18.9)	78 (32.5)	< 0.0001
Child alone can stand a few seconds without aid: *not yet*	1117	491 (44)	367 (41.8)	124 (51.7)	0.008
Child can move from standing to sitting position without dropping: *not yet*	1112	484 (43.5)	358 (41)	126 (52.9)	0.001
Child can stand up without help: *not yet*	1113	996 (89.5)	776 (88.9)	220 (91.7)	0.24
Child can creep backwards and forwards: *not yet*	1112	157 (14.1)	106 (12.2)	51 (21.3)	0.0007
Child walks along furniture while holding himself: *not yet*	1116	619 (55.5)	470 (53.7)	149 (62.1)	0.023
Child can make a few steps being held with both hands: *not yet*	1113	208 ()	141 (16.1)	67 (28)	< 0.0001
Child can make a few steps with the help of mother’s hand: *not yet*	1116	622 (55.7)	472 (53.9)	150 (62.5)	0.019
***Motor coordination***					
Child tries to catch an object by stretching his arm or body: *not yet*	1452	0.46 (0.4)	3 (0.3)	3 (1)	0.097
Child throws a ball with a forward movement of the arm: *not yet*	1450	148 (10.2)	115 (9.9)	33 (11.4)	0.45
Child can hold a little object between finger and thumb: *not yet*	1448	36 (2.5)	24 (2.1)	12 (4.2)	0.055
Child can scribble on a sheet of paper after showing him how to: *not yet*	1448	1010 (69.8)	807 (69.6)	203 (70.5)	0.77
Child can scribble on a sheet of paper without showing him how to: *not yet*	1447	76.4 (1106)	882 (76)	224 (78)	0.49
***Language***					
Says different syllables: *not yet*	1451	8 (0.6)	5 (0.4)	3 (1)	0.20
Reacts to some familiar words: *not yet*	1436	231 (16.1)	176 (15.3)	55 (19.1)	0.13
Says or repeats a word with the same 2 syllables: *not yet*	1452	49 (3.4)	34 (2.9)	15 (5.2)	0.068
Says or repeats ‘Mom’ or ‘Dad’: *not yet*	1449	129 (8.9)	92 (7.9)	37 (12.9)	0.011
Says ‘Mom’ when sees or wants mother: *not yet*	1452	596 (41)	461 (39.6)	135 (46.7)	0.032
Says ‘Dad’ when sees or wants father: *not yet*	1440	699 (48.5)	542 (47)	157 (54.7)	0.021
Says a word with 2 different syllables: *not yet*	1422	871 (61.3)	663 (58.3)	208 (73)	< 0.0001
Understands a simple order without accompanying gesture: *not yet*	1441	221 (15.3)	165 (14.3)	56 (19.6)	0.028
Shakes his head to say ‘No’: *not yet*	1444	557 (38.6)	432 (37.3)	125 (43.6)	0.058
Shows a part of own body when shown: *not yet*	1450	1193 (82.3)	945 (81.3)	248 (86.4)	0.047

^a^ N is varying because of missing answers, but for global development, most items were no more assessed as child was already walking.

^b^ Exact Fisher’s test

Scores built from summing the different items by domains and the corresponding chosen thresholds to define poorer development are described in Table 4.

**Table 4:** Score description: low scores rates according to social withdrawal status and Odds Ratios from multiple logistic models. The EDEN Mother-Child Cohort.

outcome	range	threshold	Cases N (%)	ADBB < 5 n = 1163	ADBB6 5 n = 289 (20%)	p	Adjusted OR	95% CI	P
***Motor ability***									
Midwife assessment	0 to 5	≤3	115 (7.9)	77 (6.6)	38 (13.1)	0.0006	**1.99**	**[1.29–3.07]**	**0.002**
Parental questionnaire	0 to 10	≤5	366 (25.2)	259 (22.3)	107 (37)	<0.0001	**1.93**	**[1.44–2.57]**	**< .0001**
***Coordination***									
Parental questionnaire	0 to 5	≤2	140 (9.6)	103 (8.9)	37 (12.8)	0.04	**1.35**	**[0.89–2.04]**	**0.16**
***Language***									
Midwife assessment	0 to 5	= 0	440 (30.3)	270 (23.2)	170 (58.8)	<0.0001	**5.00**	**[3.72–6.71]**	**< .0001**
Parental questionnaire	0 to 10	≤5	319 (22)	235 (20.2)	84 (29.1)	0.0007	**1.55**	**[1.13–2.12]**	**0.006**

How maternal and baby characteristics were associated with each developmental score is displayed in supplementary material (S2–S6 Tables). The rate of children under these thresholds differed significantly according to social withdrawal status. After taking into account relevant adjustment factors in multiple logistic regression models, the risk of having a lower developmental score was significantly increased in children with a high ADBB score for motor ability as assessed by the midwife (OR [95%IC] = 1.99 [1.29;3.07]), motor ability from questionnaire (OR [95%IC] = 1.93 [1.44;2.57]), clinically assessed language (OR [95%IC] = 5.00 [3.72;6.71]) and language assessed by questionnaire (OR [95%IC] = 1.55 [1.13;2.12]). No significant increased risk was observed for motor coordination as assessed with parental questionnaire (p = 0.62).

Table 5 displays the complete multivariate logistic regression model for clinically assessed language, showing that social withdrawal behaviour was one of the main predictors along with age at assessment.

**Table 5:** Multiple logistic regression model for the risk of absence of language manifestation at age 1, assessed by the midwife during clinical examination. The EDEN Mother-Child Cohort N = 1452.

	OR	95% CI	p
**Social withdrawal**	**5.0**	**[3.72–6.71]**	**<.0001**
Centre (Nancy)	0.26	[0.19–0.34]	<.0001
Female gender	1.01	[0.78–1.29]	0.96
Exact age at clinical assessment	0.98	[0.97–0.99]	0.004
Length of gestation (weeks)	0.97	[0.9–1.05]	0.46
Birth weight Z-Score	0.92	[0.82–1.03]	0.14
Maternal age at delivery (years)	1.01	[0.98–1.04]	0.42
Duration of hospitalisation during pregnancy	0.97	[0.94–1.01]	0.11
Breastfeeding duration (months)	0.99	[0.95–1.02]	0.51
Main day time caregiver:			
Nursery	0.85	[0.54–1.33]	0.69
Other	0.72	[0.53–0.97]	0.36
Family	0.65	[0.4–1.08]	0.29
Mother	1		
Maternal depression (EPDS) score at 1 year			
Unknown	1.24	[0.71–2.18]	0.74
< 10	1.31	[0.9–1.9]	0.34
≥ 10	1		
Maternal alcohol intake during pregnancy (Yes vs. No)	1.22	[0.94–1.59]	0.14
Maternal smoking during pregnancy (cigarettes/day)			
0	1.13	[0.58–2.21]	0.44
1–9	0.94	[0.46–1.9]	0.57
≥ 10	1		
Parental education* (years):			
≤ 12	1.01	[0.76–1.33]	0.97
> 12	1		

* Calculated as the average of father’s and mother’s years of education

Finally, excluding children born prematurely or with who midwifes encountered difficulties during the clinical examination did not change the results.

## Discussion

In the present study, we examined the associations between social withdrawal and some important aspects of child development, namely motor and language abilities. The results show that higher scores on social withdrawal behaviour as assessed with the ADBB scale are associated with lower scores on language outcomes, and to a lesser extent with lower scores on motor abilities.

The reason why social withdrawal behaviour is associated with lower scores in language and motor development remains to be fully understood. This may be due to the setting of the examination itself: socially withdrawn children may not engage as fully in the situation and therefore be assessed as more delayed. As such, the low scores observed by the midwife would be situation specific rather than corresponding to some actual developmental delays. However, this does not seem to be the case as we observed similar relationships with parental ratings as well. Therefore, social withdrawal behaviour in itself could be the driving force explaining these associations: socially withdrawn children are less likely to engage in stimulating activities, which are necessary for a normal developmental trajectory. Consistent with this interpretation, the study by Milne & al [19] showed that socially withdrawn infants at age 6 months displayed impaired language and communication abilities at age 2.5 years. Also, in a study by Molteno & al 2014 [20] on prenatal alcohol exposure, children later diagnosed with foetal alcohol syndrome (FAS) and partial FAS (PFAS) at 5 years exhibited more social withdrawal behaviour and less responsiveness and activity as infants. Social withdrawal in infancy might therefore lead to adverse developmental consequences. In a previous study on perinatal risk factors within the same EDEN cohort, gestational age and birth weight significantly predicted social withdrawal behaviour at age one [18]. In a follow-up analysis of the sample at age 3 and 5 years, social withdrawal independently predicted emotional regulation disorders at age 3 years and behavioural disorders at age 5 years [10]. Besides, an analysis of the predictors of Inattention-Hyperactivity showed that a low family socioeconomic status before pregnancy predicted Inattention-Hyperactivity symptoms at 3 years, with the following risk pathway: lower SES was associated with higher maternal depression and anxiety during pregnancy; then to higher maternal and child distress and dysregulation in infancy; and in turn to higher levels of inattention-hyperactivity at 3 years [21]. Similarly, gestational age/birth weight may lead to increased vulnerability to social withdrawal behaviour, which in turn may lead to more vulnerable peer relationships, less psychomotor and language abilities, less efficient emotional regulation at age 3, and more behavioural difficulties at age 5. In the same EDEN sample, Peyre &al have shown which are the elements predicting language skills between 2 and 3 years of age [22]. Future studies should further investigate the role of social withdrawal behaviour in this putative developmental pathway. However, this study suggests that social withdrawal behaviour could be a mediator between perinatal risk and later psychopathology, even for young children, as it seems to be the case for older ones [23, 24].

### Strengths and limitations

One limitation of the study is the possible bias in the selection of the sample: because of the area-based mode of recruitment and the selective acceptation to participate in the study, urban, well-educated and high-income households are over represented in the EDEN mothers compared to the national population [9]. As a result, the range of socioeconomic circumstances in our study is not as wide as in the general population, and the association between socioeconomic status and children’s behavior may be stronger than we report. This characteristic was further reinforced by a higher proportion lost to-follow-up at one year among poorer and less educated families. Besides, the attrition rate, though acceptable, slightly reduces the power of the study. The study centre was a main predictor of developmental scores; this may reflect some inter examination differences as well as some unmeasured socioeconomic factors that could differ between the two cities; but whatever the source of variability, this was, at least in part, taken into account in the analyses by adjusting for centre.

Another limitation is that the assessment of psychomotor and language abilities was made by a combination of items from two different scales, from which we built our own scores, and that did not provide a comprehensive evaluation of cognitive skills. Indeed, we could not implement long psychomotor evaluations within the one-year examination of the EDEN study, as the duration of the examination was limited to one hour, with a wide range of clinical assessments to be made. That is why we retained from the Brunet-Lézine only the items that could be observed by the midwife. Concerning the Bayley, no validated French version has been proposed yet; that made us impossible to use existing validated score calculation. The age at assessment was not itself corrected for gestational age in preterm children, but gestational age was included in the multivariable model, and therefore taken into account in the analysis. In fact, excluding all premature babies (<37 weeks) from the analysis did not change the results.

The cross-sectional design of the present study makes it impossible to make any causal interpretation of the observed associations. Intervention studies focussing directly on social withdrawal behaviour and assessing the consequences of such intervention on psychomotor and language development may be necessary for a better understanding of the potential causal role played by social withdrawal behaviour in infants. Individual differences between levels of development at this age are indeed quite large. However, the language and psychomotor development in this large sample appear to be in the normal range with most relevant milestones being in the 90th percentile for age [25]. Another point lies in that most 12-month-olds have not started to speak and therefore it is not clear what the language items in the study effectively measure. Those items may in fact reflect communication abilities, or be linked to the child's introversion and extroversion (i.e. temperament) rather than to social withdrawal behavior in itself. As 4 out of 8 items in the ADBB appear to be partly linked with temperament, co–variation is inherent in the measures of both language milestones and social withdrawal behavior, and in a lesser degree, between social withdrawal behavior and psychomotor milestones. In two recent controlled studies social withdrawal behavior and temperament appear as two different, though overlapping dimensions [26, 27].

The strengths of the study are the size of the sample and the control for multiple environmental social and relational confounding factors. In addition, social withdrawal was assessed during a paediatric routine examination by midwifes, and both midwives and parents independently assessed developmental outcomes, thereby limiting the possibility that our results arose solely from common method variance.

Being socially withdrawn at 1 year is associated with delays in language and in a lesser extent, with lower psychomotor milestones in the child, in this low risk, voluntary sample. This may have important consequences for a better understanding of the mutual influences of the several dimensions of early development (i.e.: psychomotor, language, and intersubjective development). This also calls for early screening of social withdrawal behaviour in infants.

## Supporting Information

S1 Table Maternal and infant characteristics between children included or not in the analysis. (DOCX)

S2 Table Maternal and infant characteristics according to score of motor ability assessed by the midwife. (DOCX)

S3 Table Maternal and infant characteristics according to score of motor ability assessed by asking the mother. (DOCX)

S4 Table Maternal and infant characteristics according to score of coordination assessed by questioning the mother. (DOCX)

S5 Table Maternal and infant characteristics according to score of language assessed by the midwife. (DOCX)

S6 Table Maternal and infant characteristics according to score of language assessed by questioning the mother. (DOCX)
