# Supplementary material for: Social Withdrawal Behaviour at One Year of Age Is Associated with Delays in Reaching Language Milestones in the EDEN Mother-Child Cohort Study
Source: PLoS One. 2016 Jul 8;11(7):e0158426. doi: 10.1371/journal.pone.0158426 (PMC4938506; doi:10.1371/journal.pone.0158426)
Supplement: S1 Table — (DOCX) [file pone.0158426.s001.docx]

**Supplementary Table 1**: Maternal and infant characteristics between children included or not in the analysis.

|  | **Not included**  **N=455** | **Included**  **N=1452** | p |
| --- | --- | --- | --- |
| Centre (Nancy) | 225 (49.5) | 740 (51) | 0,57 |
| Male gender | 233 (51.7) | 767 (52.8) | 0,67 |
| Length of gestation (weeks) | 39.1 ± 0.08 | 39.26 ± 0.05 | 0,076 |
| Birth weight z-score (Gardosi) | -0.09 ± 0.05 | -0.02 ± 0.03 | 0,26 |
| Maternal age at delivery (years) | 28.63 ± 0.23 | 29.75 ± 0.13 | <.0001 |
| Hospitalisation during pregnancy (days) | 1.37 ± 0.21 | 1.33 ± 0.12 | 0,85 |
| Duration of breastfeeding (months) | 2.54 ± 0.17 | 3.4 ± 0.09 | <.0001 |
| Maternal alcohol intake during pregnancy | 196 (43.6) | 642 (44.2) | 0,81 |
| Maternal smoking during pregnancy (cigarettes/day): 0 | 256 (56.9) | 1113 (76.7) | <.0001 |
| 1-9 | 152 (33.8) | 287 (19.8) | . |
| ≥ 10 | 42 (9.3) | 52 (3.6) | . |
| Parental education* (years): ≤ 12 | 233 (51.4) | 558 (38.4) | <.0001 |
| > 12 | 220 (48.6) | 894 (61.6) | . |

Numbers are N (%) or m ± sd

*Calculated as the average of father’s and mother’s years of education
