# Supplementary material for: Social Withdrawal Behaviour at One Year of Age Is Associated with Delays in Reaching Language Milestones in the EDEN Mother-Child Cohort Study
Source: PLoS One. 2016 Jul 8;11(7):e0158426. doi: 10.1371/journal.pone.0158426 (PMC4938506; doi:10.1371/journal.pone.0158426)
Supplement: S2 Table — (DOCX) [file pone.0158426.s002.docx]

**Supplementary Table 2**: Maternal and infant characteristics according to score of motor ability assessed by the midwife.

|  | **Low score**  **N=115** | **Others**  **N=1337** | p |
| --- | --- | --- | --- |
| Centre (Nancy) | 49 (42.6) | 691 (51.7) | 0.062 |
| Male gender | 53 (46.1) | 714 (53.4) | 0.13 |
| Exact age of the child at examination (days) | 366.7 ± 1.1 | 370.4 ± 0.3 | 0.001 |
| Length of gestation (weeks) | 38.8 ± 0.2 | 39.3 ± 0 | 0.0008 |
| Birth weight z-score (Gardosi) | -0.2 ± 0.1 | 0 ± 0 | 0.096 |
| Maternal age at delivery (years) | 29.8 ± 0.4 | 29.7 ± 0.1 | 0.93 |
| Hospitalisation during pregnancy (days) | 2.3 ± 0.4 | 1.2 ± 0.1 | 0.014 |
| Duration of breastfeeding (months) | 2.9 ± 0.4 | 3.4 ± 0.1 | 0.16 |
| Main mode of day care : Nursery | 22 (19.1) | 147 (11) | 0.024 |
| Other | 46 (40) | 560 (41.9) | . |
| Family | 5 (4.3) | 131 (9.8) | . |
| Mother | 42 (36.5) | 499 (37.3) | . |
| Maternal EPDS depression score at 1 year:  Unknown | 6(5.2) | 98 (7.3) | 0.21 |
| < 10 | 98(85.2) | 1046 (78.2) | . |
| ≥ 10 | 11 (9.6) | 193 (14.4) | . |
| Maternal alcohol intake during pregnancy (yes) | 51 (44.3) | 591 (44.2) | 0.98 |
| Maternal smoking during pregnancy (cigarettes/day): 0 | 90 (78.3) | 1023 (76.5) | 0.91 |
| 1-9 | 21 (18.3) | 266 (19.9) | . |
| ≥ 10 | 4 (3.5) | 48 (3.6) | . |
| Parental education* (years): > 12 | 68 (59.1) | 826 (61.8) | 0.57 |

Numbers are N (%) or m ± SD

*Calculated as the average of father’s and mother’s years of education
