# Supplementary material for: Social Withdrawal Behaviour at One Year of Age Is Associated with Delays in Reaching Language Milestones in the EDEN Mother-Child Cohort Study
Source: PLoS One. 2016 Jul 8;11(7):e0158426. doi: 10.1371/journal.pone.0158426 (PMC4938506; doi:10.1371/journal.pone.0158426)
Supplement: S3 Table — (DOCX) [file pone.0158426.s003.docx]

**Supplementary Table 3**: Maternal and infant characteristics according to score of motor ability assessed by asking the mother.

|  | **Low score**  **N=366** | **Others**  **N=1086** | p |
| --- | --- | --- | --- |
| Centre (Nancy) | 168 (45.9) | 572 (52.7) | 0.025 |
| Male gender | 179 (48.9) | 588 (54.1) | 0.083 |
| Exact age of the child at examination (days) | 367.8 ± 0.6 | 370.9 ± 0.4 | <.0001 |
| Length of gestation (weeks) | 38.9 ± 0.1 | 39.4 ± 0.1 | <.0001 |
| Birth weight z-score (Gardosi) | -0.1 ± 0.1 | 0 ± 0 | 0.24 |
| Maternal age at delivery (years) | 30.3 ± 0.3 | 29.6 ± 0.1 | 0.014 |
| Hospitalisation during pregnancy (days) | 1.9 ± 0.2 | 1.1 ± 0.1 | 0.006 |
| Duration of breastfeeding (months) | 3 ± 0.2 | 3.5 ± 0.1 | 0.014 |
| Main mode of day care : Nursery | 52 (14.2) | 117 (10.8) | 0.009 |
| Other | 165 (45.1) | 441 (40.6) | . |
| Family | 21 (5.7) | 115 (10.6) | . |
| Mother | 128 (35) | 413 (38) | . |
| Maternal EPDS depression score at 1 year:  Unknown | 20 (5.5) | 84 (7.7) | 0.34 |
| < 10 | 295 (80.6) | 849 (78.2) | . |
| ≥ 10 | 51 (13.9) | 153 (14.1) | . |
| Maternal alcohol intake during pregnancy (yes) | 170 (46.4) | 472 (43.5) | 0.32 |
| Maternal smoking during pregnancy (cigarettes/day): 0 | 280 (76.5) | 833 (76.7) | 0.99 |
| 1-9 | 73 (19.9) | 214 (19.7) | . |
| ≥ 10 | 13 (3.6) | 39 (3.6) | . |
| Parental education* (years): > 12 | 225 (61.5) | 669 (61.6) | 0.97 |

Numbers are N (%) or m ± SD

*Calculated as the average of father’s and mother’s years of education
