# Supplementary material for: Social Withdrawal Behaviour at One Year of Age Is Associated with Delays in Reaching Language Milestones in the EDEN Mother-Child Cohort Study
Source: PLoS One. 2016 Jul 8;11(7):e0158426. doi: 10.1371/journal.pone.0158426 (PMC4938506; doi:10.1371/journal.pone.0158426)
Supplement: S4 Table — (DOCX) [file pone.0158426.s004.docx]

**Supplementary Table 4**: Maternal and infant characteristics according to score of coordination assessed by questioning the mother.

|  | **Low score**  **N=140** | **Others**  **N=1312** | p |
| --- | --- | --- | --- |
| Centre (Nancy) | 74 (52.9) | 666 (50.8) | 0.64 |
| Male gender | 73 (52.1) | 694 (52.9) | 0.87 |
| Exact age of the child at examination (days) | 369.2 ± 1 | 370.2 ± 0.3 | 0.32 |
| Length of gestation (weeks) | 38.6 ± 0.1 | 39.3 ± 0 | <.0001 |
| Birth weight z-score (Gardosi) | 0 ± 0.1 | 0 ± 0 | 0.81 |
| Maternal age at delivery (years) | 30.4 ± 0.4 | 29.7 ± 0.1 | 0.11 |
| Hospitalisation during pregnancy (days) | 2 ± 0.4 | 1.3 ± 0.1 | 0.07 |
| Duration of breastfeeding (months) | 3.2 ± 0.3 | 3.4 ± 0.1 | 0.47 |
| Main mode of day care: Nursery | 19 (13.6) | 150 (11.4) | 0.80 |
| Other | 60 (42.9) | 546 (41.6) | . |
| Family | 11 (7.9) | 125 (9.5) | . |
| Mother | 50 (35.7) | 491 (37.4) | . |
| Maternal EPDS depression score at 1 year:  Unknown | 9 (6.4) | 95 (7.2) | 0.03 |
| < 10 | 101 (72.1) | 1043 (79.5) | . |
| ≥ 10 | 30 (21.4) | 174 (13.3) | . |
| Maternal alcohol intake during pregnancy (yes) | 71 (50.7) | 571 (43.5) | 0.10 |
| Maternal smoking during pregnancy (cigarettes/day): 0 | 113 (80.7) | 1000 (76.2) | 0.17 |
| 1-9 | 20 (14.3) | 267 (20.4) | . |
| ≥ 10 | 7 (5) | 45 (3.4) | . |
| Parental education* (years): > 12 | 91 (65) | 803 (61.2) | 0.38 |

Numbers are N (%) or m ± SD

*Calculated as the average of father’s and mother’s years of education
