# Supplementary material for: Social Withdrawal Behaviour at One Year of Age Is Associated with Delays in Reaching Language Milestones in the EDEN Mother-Child Cohort Study
Source: PLoS One. 2016 Jul 8;11(7):e0158426. doi: 10.1371/journal.pone.0158426 (PMC4938506; doi:10.1371/journal.pone.0158426)
Supplement: S5 Table — (DOCX) [file pone.0158426.s005.docx]

**Supplementary Table 5**: Maternal and infant characteristics according to score of language assessed by the midwife.

|  | **Low score**  **N=440** | **Others**  **N=1012** | p |
| --- | --- | --- | --- |
| Centre (Nancy) | 129 (29.3) | 611 (60.4) | <.0001 |
| Male gender | 239 (54.3) | 528 (52.2) | 0.45 |
| Exact age of the child at examination (days) | 367.9 ± 0.6 | 371.1 ± 0.4 | <.0001 |
| Length of gestation (weeks) | 39.2 ± 0.1 | 39.3 ± 0.1 | 0.28 |
| Birth weight z-score (Gardosi) | -0.1 ± 0.1 | 0 ± 0 | 0.37 |
| Maternal age at delivery (years) | 29.8 ± 0.2 | 29.7 ± 0.2 | 0.86 |
| Hospitalisation during pregnancy (days) | 1.3 ± 0.2 | 1.4 ± 0.1 | 0.69 |
| Duration of breastfeeding (months) | 3.1 ± 0.2 | 3.5 ± 0.1 | 0.033 |
| Main mode of day care : Nursery | 44 (10) | 125 (12.4) | 0.005 |
| Other | 173 (39.3) | 433 (42.8) | . |
| Family | 31 (7) | 105 (10.4) | . |
| Mother | 192 (43.6) | 349 (34.5) | . |
| Maternal EPDS depression score at 1 year:  Unknown | 43 (9.8) | 6 (61) | 0.04 |
| < 10 | 337 (76.6) | 807 (79.7) | . |
| ≥ 10 | 60 (13.6) | 144 (14.2) | . |
| Maternal alcohol intake during pregnancy (yes) | 210 (47.7) | 432 (42.7) | 0.076 |
| Maternal smoking during pregnancy (cigarettes/day): 0 | 337 (76.6) | 776 (76.7) | 0.77 |
| 1-9 | 85 (19.3) | 202 (20) | . |
| ≥ 10 | 18 (4.1) | 34 (3.4) | . |
| Parental education* (years): > 12 | 250 (56) | 644 (63.6) | 0.014 |

Numbers are N (%) or m ± SD

*Calculated as the average of father’s and mother’s years of education
