# Supplementary material for: Social Withdrawal Behaviour at One Year of Age Is Associated with Delays in Reaching Language Milestones in the EDEN Mother-Child Cohort Study
Source: PLoS One. 2016 Jul 8;11(7):e0158426. doi: 10.1371/journal.pone.0158426 (PMC4938506; doi:10.1371/journal.pone.0158426)
Supplement: S6 Table — (DOCX) [file pone.0158426.s006.docx]

**Supplementary Table 6**: Maternal and infant characteristics according to score of language assessed by questioning the mother.

|  | **Low score**  **N=319** | **Others**  **N=1133** | p |
| --- | --- | --- | --- |
| Centre (Nancy) | 84 (26.3) | 656 (57.9) | <.0001 |
| Male gender | 195 (61.1) | 572 (50.5) | 0.0008 |
| Exact age of the child at examination (days) | 368.3 ± 0.6 | 370.7 ± 0.3 | 0.001 |
| Length of gestation (weeks) | 39.2 ± 0.1 | 39.3 ± 0.1 | 0.59 |
| Birth weight z-score (Gardosi) | -0.1 ± 0.1 | 0 ± 0 | 0.27 |
| Maternal age at delivery (years) | 30.8 ± 0.3 | 29.5 ± 0.1 | <.0001 |
| Hospitalisation during pregnancy (days) | 1.2 ± 0.3 | 1.3 ± 0.1 | 0.73 |
| Duration of breastfeeding (months) | 3.2 ± 0.2 | 3.5 ± 0.1 | 0.23 |
| Main mode of day care : Nursery | 34 (10.7) | 135 (11.9) | 0.23 |
| Other | 148 (46.4) | 458 (40.4) | . |
| Family | 24 (7.5) | 112 (9.9) | . |
| Mother | 113 (35.4) | 428 (37.8) | . |
| Maternal EPDS depression score at 1 year:  Unknown | 29 (9.1) | 75 (6.6) | 0.31 |
| < 10 | 247 (77.4) | 897 (79.2) | . |
| ≥ 10 | 43 (13.5) | 161 (14.2) | . |
| Maternal alcohol intake during pregnancy (yes) | 182 (57.1) | 460 (40.6) | <.0001 |
| Maternal smoking during pregnancy (cigarettes/day): 0 | 260 (81.5) | 853 (75.3) | 0.063 |
| 1-9 | 51 (16) | 236 (20.8) | . |
| ≥ 10 | 8 (2.5) | 44 (3.9) | . |
| Parental education* (years): > 12 | 207 (64.9) | 687 (60.6) | 0.168 |

Numbers are N (%) or m ± SD

*Calculated as the average of father’s and mother’s years of education
